# Fabrication and Gas-Sensing Properties of Ni-Silicide/Si Nanowires

**DOI:** 10.1186/s11671-017-1955-6

**Published:** 2017-03-09

**Authors:** Hsun-Feng Hsu, Chun-An Chen, Shang-Wu Liu, Chun-Kai Tang

**Affiliations:** Department of Materials Science and Engineering, National Chung Hsing University, 145 Xingda Rd., Taichung, 40227 Taiwan

**Keywords:** Nanowire, Sensor, Si, Silicide, Schottky barrier, Scanning probe lithography

## Abstract

Ni-silicide/Si nanowires were fabricated by atomic force microscope nano-oxidation on silicon-on-insulator substrates, selective wet etching, and reactive deposition epitaxy. Ni-silicide nanocrystal-modified Si nanowire and Ni-silicide/Si heterostructure multi-stacked nanowire were formed by low- and high-coverage depositions of Ni, respectively. The Ni-silicide/Si Schottky junction and Ni-silicide region were attributed high- and low-resistance parts of nanowire, respectively, causing the resistance of the Ni-silicide nanocrystal-modified Si nanowire and the Ni-silicide/Si heterostructure multi-stacked nanowire to be a little higher and much lower than that of Si nanowire. An O_2_ sensing device was formed from a nanowire that was mounted on Pt electrodes. When the nanowires exposed to O_2_, the increase in current in the Ni-silicide/Si heterostructure multi-stacked nanowire was much larger than that in the other nanowires. The Ni-silicide nanocrystal-modified Si nanowire device had the highest sensitivity. The phenomenon can be explained by the formation of a Schottky junction at the Ni-silicide/Si interface in these two types of Ni-Silicide/Si nanowire and the formation of a hole channel at the silicon nanowire/native oxide interface after exposing the nanowires to O_2_.

## Background

Semiconductor nanowires (NWs) are ideal components of sensing devices because of their high surface-to-volume ratio [1–4].

Gas sensors that are based on Si nanostructures are emerging as very powerful because they are readily compatible with existing semiconductor processing technologies. In previous studies, Si nanostructures have been used to detect different gas molecules (such as NH_3_, NO, H_2_, O_2_, and NO_2_) [5]. The gas-sensing SiNW-based devices consist of both vertically standing [6–8] and in-plane-orientated SiNWs [9–15]. In-plane-orientated SiNW-based devices are more easily integrated into multifunction devices than are vertically standing SiNW-based ones. In-plane-orientated SiNW-based devices can be fabricated using two well-known methods. In the first, nanowires are grown by bottom-up fabrication or etching methods and then transferred onto a substrate; contact pads are then fabricated [9–11]. The second method is a top-down method in which large-scale patterns are formed on a substrate and the lateral dimensions reduced to the nanoscale by nanoimprinting [12–14] or electron-beam lithography [15]; this method has the advantages of both reproducibility and reliability. In this work, the nanowires were fabricated by atomic force lithography and selective wet etching on a silicon-on-insulator (SOI) wafer, before the contact pads were fabricated.

With respect to in-plane-orientated nanowire-based devices, the sensing performance of metal oxide semiconductor nanowire is enhanced by functionalization with metal or semiconductor nanoparticles, because of the formation of nanosized Schottky or p-n junctions, which forms electron depletion regions within the nanowire, shrinking the effective conduction channel and effectively manipulating the local charge carrier concentration [16–18].

Silicide/Si heterostructures are widely used for source/drain contacts and the low-resistivity interconnects of conventional silicon devices. They have been incorporated into Si nanowires. Nickel silicide/silicon contacts in silicon nanowires are Schottky junctions [19, 20] and can be used to fabricate multifunctional devices [21, 22].

In this work, a Ni-silicide nanocrystal was used to functionalize the Si nanowires, and the O_2_ sensing performance of the Si nanowires with Ni-silicide nanocrystals was compared with that of those without.

## Methods

**Fig. 1:**
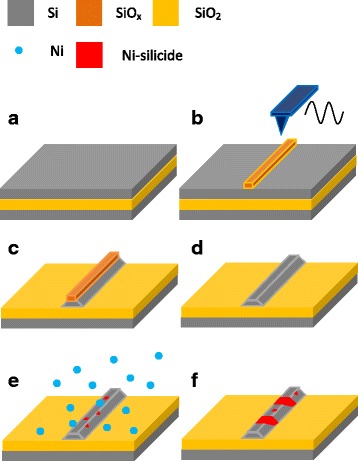
Schematic diagrams of sample preparation. **a** Cleaned SOI substrate. **b** AFM oxidation. **c** Selected etching by KOH solution. **d** Selected etching by HF solution. **e** Ni deposition at elevated temperature. **f** Unreactive Ni atom removing by HNO_3_ solution

Figure 1 shows the process by which Ni-silicide nanocrystal-functionalized silicon nanowires are fabricated. SOI wafers that were fabricated by the Smart-Cut process were used. A p-type silicon top layer with a thickness of 55 nm and a resistance of about 0.12 Ω cm was insulated from the bulk silicon substrate by a 150-nm-thick buried oxide. The silicon layers were cleaned by a standard RCA process [23] and, then, dipped in a dilute HF solution before being nano-oxidized by atomic force microscopy (AFM). Nano-oxidation was performed using a commercial AFM (Solver PRO-M, NT-MDT) that was operated in the tapping mode using PtIr-coated silicon tips (under a constant force of about 3.3 N/m, at a resonance frequency of about 88 kHz). The Si nanowires were formed by immersing the nano-oxidized samples in the 20 wt% KOH solution at 50 °C for 40 s. The samples were dipped in a dilute HF solution to remove the native oxide layer on the surface of Si nanowires and immediately loaded into an ultra-high vacuum (UHV) mini-e-beam deposition chamber with a base pressure about 5 × 10^−10^ torr. Next, Ni films were deposited with the deposition rate of 0.01 Å/s at 400 °C. The deposition pressure was approximately 1 × 10^−9^ torr. Thereafter, the sample temperature was held at 400 °C for 1 h. The unreactive Ni atoms were removed using HNO_3_ solution. Al/Ti electrodes were fabricated using shadow mask on the substrate, and the two ends of the nanowire were fixed by the deposition of Pt using a focused ion beam. Finally, the formed devices were annealed at 300 °C for 30 s.

For O_2_ sensing experiments, nanowire device was placed in a vacuum furnace tube with the base pressure of 1 × 10^−3^ torr, in which the flux of the gases could be controlled. The sensitivity (*S*) was calculated as Δ*I*/*I*_0_, where Δ*I* is the change in current that is induced by exposure of the device to the target gas atmosphere and *I*_0_ is the initial current.

## Results and Discussion

**Fig. 2:**
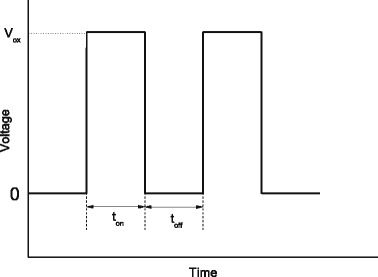
Nomenclature for an AFM oxidation voltage pulse

**Fig. 3:**
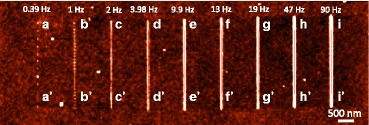
AFM image of the oxide lines that are written on the SOI substrate with various oxidation frequencies at a sample voltage of 40 V and a scan speed of 0.1 μm/s

Figure 2 shows the control parameters for a periodic DC pulse (exposure time, *t*_on_, release time, *t*_off_, and oxidation voltage, *V*_ox_). The oxidation frequency, *f*, is defined as (*t*_on_ + *t*_off_)^−1^. The exposure time is fixed at 1 ms, and various oxidation frequencies are used with different release times. Figure 3 shows the oxide lines that are written on the SOI substrate in the Si < 110 > direction with various oxidation frequencies at a sample voltage of 40 V and a scan speed of 0.1 μm/s. The temperature is room temperature (25 ± 2 °C). Samples were put in a dry box, and the relative humidity is controlled at 45 ± 1%.

**Fig. 4:**
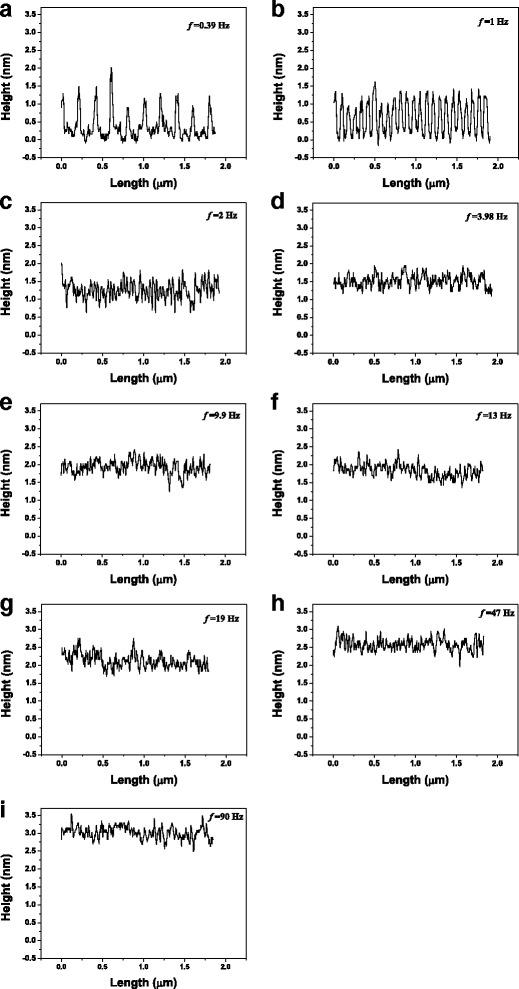
The image brightness profiles along the (**a**) aa′, (**b**) bb′, (**c**) cc′, (**d**) dd′, (**e**) ee′, (**f**) ff′, (**g**) gg′, (**h**) hh′, and (**i**) ii′ are shown in Fig. 3, in which the average height of the substrate is 0 nm, for the oxide lines written with 0.39, 1, 2, 3.98, 9.9, 13, 19, 47, and 90 Hz, respectively

**Fig. 5:**
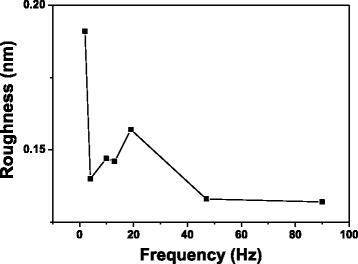
Roughness of oxide lines with various oxide frequencies (≥2 Hz). The roughness of the oxide lines is the roughness average, Ra, of the line profiles in Fig. 4c–i

**Fig. 6:**
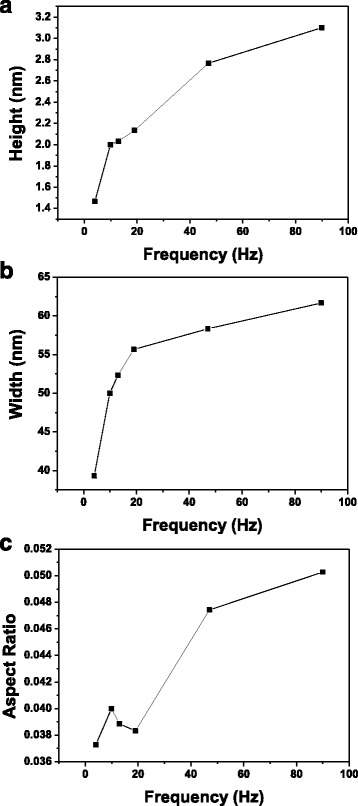
The **a** height, **b** width, and **c** aspect ratio of the oxide line written with various oxide frequencies (≥3.98 Hz). *Note*: **a** is the average magnitude of the height of line profiles shown in Fig. 4d–i

Figure 4 plots the line profiles of the oxide lines indicating that when oxide frequency ≤1 Hz, the fluctuation in the height of the oxide line was periodic because the oxide protrusions were separated from each other by the application of periodic DC pulse power. The periodic fluctuation in the height of oxide line was not obvious at oxide frequency ≥2 Hz, indicating that the oxide protrusions overlapped. The relation between the roughness of the oxide line vs. oxide frequency, as shown in Fig. 5, shows that when frequency ≥3.98 Hz, the roughness was lower than when the oxide frequency = 2 Hz because a continuous uniform oxide line formed. Figure 6 plots the relationships between the height, width, and aspect ratio of oxide lines and the frequency of the DC pulse power. The height and width of the oxide line increased with the frequency.

**Fig. 7:**
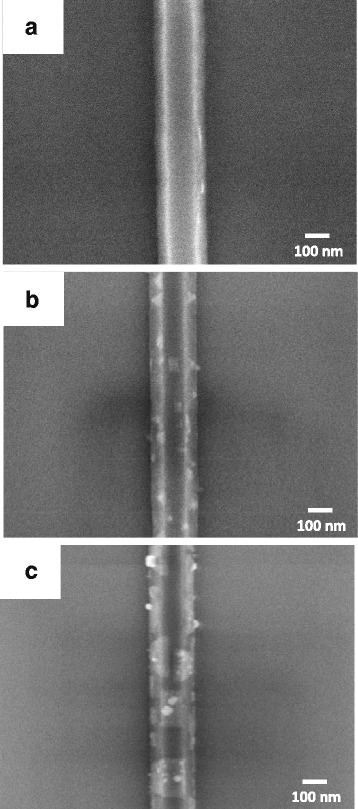
SEM images of **a** Si nanowire and Si nanowires after reacting with Ni atoms that deposited with the Ni/Si atomic ratio of **b** 1/80 and **c** 1/8 at 400 °C

Selective etching was performed by immersing the nano-oxidized sample in KOH solution. Since the ideal etching ratio of Si{100}:Si{111}:SiO_2_ is 450:1.125:1, a Si nanowire with {111}-plane sidewalls can be obtained [24, 25]. In this work, although the etching rate of the SPM oxide (SiO_*x*_) is higher than that of the thermal oxide (SiO_2_), it is still much lower than that of the Si{100}. In order to form a continuous Si wire, a high enough oxide line is required [26]. In this work, the etching time of the 55-nm-thick Si layer was about 40 s using 20 wt% KOH solution at 50 °C. The height of oxide line must exceed 2 nm. A continuous Si wire was obtained by 40 s of selective etching, as shown in Fig. 7a. Thus, frequency ≥19 Hz was selected.

Then, Ni-silicide nanocrystals were formed on Si nanowires by reactive epitaxy to deposit Ni atoms at 400 °C with various Ni/Si atomic ratios. The Ni/Si atomic ratio was estimated based on only Ni film on top of the Si nanowire reacting with Si [26]. Figure 7b, c shows SEM images of Si nanowires after their reaction with Ni atoms with Ni/Si atomic ratios of 1/80 and 1/8, respectively. A nanocrystal-modified Si nanowire (NMSiNW) was formed when the Ni/Si atomic ratio was 1/80, as shown in Fig. 7b. The size of Ni-silicide nanocrystals was smaller than 100 nm. When the Ni/Si atomic ratio was increased to 1/8, the size of the Ni-silicide crystals were increased, forming Ni-silicide/Si multi-stacked heterostructure nanowire (MSHNW), as shown in Fig. 7c.

Three devices that were based on Ni-silicide nanocrystal-modified Si nanowire, Ni-silicide/Si multi-stacked heterostructure nanowire, and Si nanowire were carried out in a vacuum chamber with a base pressure of 1 × 10^−3^ torr. The current was measured using a bias voltage of 10 V. When the temperature was increased to 250 °C, the current increased gradually, reaching a stable value after about 1 h of holding at that temperature. This phenomenon indicates that when the nanowire was in air, water condensed on its surface revealing in turn that the p-type Si nanowire exhibited high resistance [27]. When the nanowire was placed in the vacuum and heated, the water that condensed on its surface was desorbed. Thus, the resistance of the nanowire decreased, causing the current to increase. After 1 h, all of the water was desorbed and the current reached a stable value.

The resistances of Si nanowire, Ni-silicide nanocrystal-modified Si nanowire, and Ni-silicide/Si multi-stacked heterostructure nanowire at 250 °C in vacuum were 2 × 10^10^, 2.5 × 10^10^, and 3.3 × 10^9^ Ω, respectively. The resistances of the Ni-silicide nanocrystal-modified Si nanowire and Ni-silicide multi-stacked heterostructure nanowire were a little higher and much lower than that of the Si nanowire, for the following reason. Since the Ni-silicide grew into Si nanowire, two factors affect the resistance of that nanowire. One is the formation of Ni-silicide/Si interface with a Schottky characteristic, which can increase the resistance of nanowire. Furthermore, the formation of the nanoscopic depletion region in the Schottky junction reduces the cross-sectional area for carrier transmission in the Si region, increasing the resistance of nanowire. The other factor is that nanowire contains a low resistance Ni-silicide phase. Since the resistivity of Ni-silicide is much lower than that of Si, according to the resistivity-mixture rule, the resistance of the nanowire can be reduced. Thus, in the Ni-silicide nanocrystal-modified Si nanowire, the former factor has a greater effect on the resistance of nanowire than the latter factor. However, the amount of the Ni-silicide phase in the multi-stacked heterostructure nanowire is much more than that in the Ni-silicide nanocrystal-modified Si nanowire. The latter factor dominates.

**Fig. 8:**
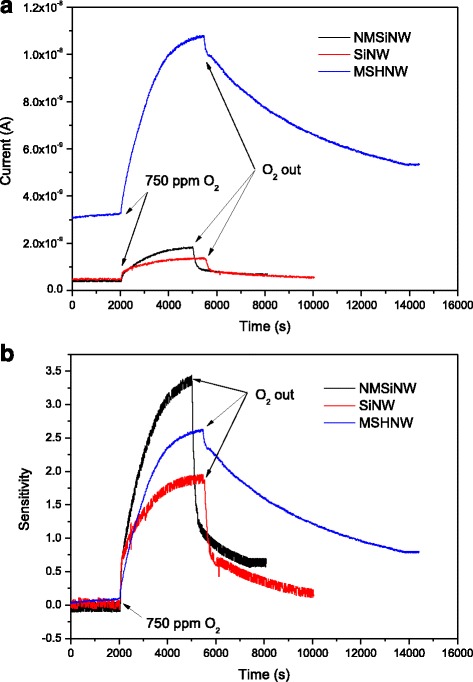
**a** Current and **b** sensitivity of the oxygen detection recorded at 250 °C for the Si nanowire (SiNW), Ni-silicide nanocrystal-modified Si nanowire (NMSiNW), and Ni-silicide/Si multi-stacked heterostructure nanowire (MSHNW)

Figure 8 shows that as the duration of exposure to oxygen, the measured current increased rapidly at first and then slowly until reaching a stable value. Similarly, when the oxygen was pumped out, the current recovered rapidly at first and then slowly. The increase and recovery of the current at two rates are attributed to a native oxide (SiO_*x*_, *x* < 2) on the nanowire surface. When oxygen was injected in vacuum chamber, O^−^ adsorbed on the native oxide surface at first, the current increased rapidly, and, then, O^−^ diffused into the native oxide layer, causing the current to increase slowly. Similarly, fast desorption of O^−^ on the native oxide surface and its diffusion out from the native oxide layer caused the fast and then slow recovery of current, respectively, when the oxygen was pumped out of the chamber.

**Fig. 9:**
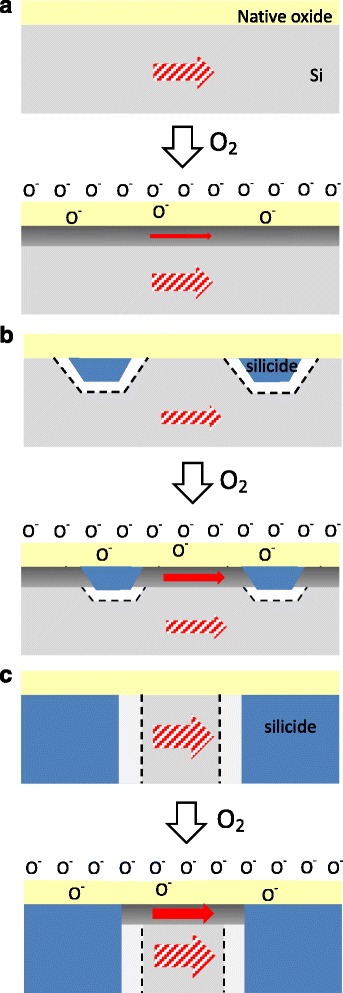
Schematic diagram of the working principle of different nanowire devices before and after being exposed to O_2_. **a** Si nanowire. **b** Ni-silicide nanocrystal-modified Si nanowire. **c** Ni-silicide/Si multi-stacked heterostructure nanowire

A schematic description of the sensing mechanism of three types of nanowire-based O_2_ sensors is depicted in Fig. 9. The increase in current in the wire can be attributed to the absorption of oxygen species on the nanowire, resulting in a net negative charge on its surface as a molecular gate. Thus, holes aggregated at the silicon nanowire/native-oxide interface and formed a hole channel, effectively reducing its resistance. However, an increase in the hole concentration reduces the energy barrier at Ni-silicide/Si interface. Thus, the increase in current that is achieved using Ni-silicide nanocrystal-modified Si nanowire was greater than those achieved using Si nanowire. When the Ni/Si ratio to 1/8 was further increased, forming Ni-silicide/Si multi-stacked heterostructure nanowire, the change in current further increased, as shown in Table 1. The initial current in Ni-silicide/Si multi-stacked heterostructure nanowire was much larger than that in nanocrystal-modified Si nanowire. That is a probable reason for the sensitivity drop (Fig. 8b) by using from nanocrystal-modified Si NW to Ni-silicide/Si multi-stacked heterostructure nanowire.

**Table 1:** The change in current (∆*I*) and sensitivity (*S*) of Si nanowire (SiNW), Ni-silicide nanocrystal modified Si nanowire (NMSiNW), and Ni-silicide/Si multi-stacked heterostructure nanowire (MSHNW) devices exposed to 750 ppm O_2_

	∆*I* (A)	*S*
Si NW	0.9 × 10^−9^	1.8
NMSiNW	1.4 × 10^−9^	3.5
MSHNW	7.5 × 10^−9^	2.27

However, the sensitivity of the nanowires may be influenced when exposed in O_2_ that mix with water. Previous reports show that the resistance of Si nanowire change when Si nanowire was operated in ambient air with various relative humidity [27] or with various pH values [28]. Furthermore, the influence decreases with the increase of temperature [27]. In this work, O_2_ sensing is operated at 250 °C. Therefore, we infer that the influence of H_2_O on the sensitivity when operating in 250 °C may be slighter than that when operating in room temperature.

## Conclusions

Si nanowires were fabricated by AFM nano-oxidation on silicon-on-insulator substrates and selective wet etching. Ni-silicide nanocrystal-modified Si nanowire and Ni-silicide/Si heterostructure multi-stacked nanowire were formed by depositing Ni with Ni/Si atomic ratio of 1/80 and 1/8, respectively, at 400 °C on Si nanowires. Since the Ni-silicide grew into Si nanowire, the Ni-silicide/Si Schottky junction and Ni-silicide region were attributed high- and low-resistance parts of nanowire, respectively, causing the resistance of the Ni-silicide nanocrystal-modified Si nanowire and the Ni-silicide/Si heterostructure multi-stacked nanowire to be a little higher and much lower than that of the Si nanowire. An O_2_ sensing device was formed from a nanowire that was mounted on Pt electrodes. The change in current in Ni-silicide/Si nanowire increases with the amount of Ni-silicide nanocrystal in the Si nanowire after the exposure of the nanowire to O_2_. The Ni-silicide nanocrystal-modified Si nanowire device had the highest sensitivity. The phenomenon can be explained by the formation of a Schottky junction at the Ni-silicide/Si interface in the Ni-Silicide/Si nanowires and the formation of a hole channel at the silicon nanowire/native oxide interface after exposing nanowires to O_2_.

To the author’s knowledge, no prior work has been reported on the gas-sensing properties of Ni-silicide/Si nanowires. More work is required to guarantee selectivity, long-time reliability, and stability in the Ni-silicide/Si nanowires.
